# Association of Superintendents of American Institutions for the Insane

**Published:** 1848

**Authors:** 


### Part 4.—Editorial.

### ARTICLE I.

### ASSOCIATION OF SUPERINTENDENTS OF AMERICAN INSTITUTIONS FOR THE INSANE.

Believing that it will be interesting to our readers, we publish entire the following account of the Proceedings of the Third Meeting of the Association of Medical Superintendents of American Institutions for the Insane, transmitted by the Secretary Dr. Kirkbride.

The Association of Medical Superintendents of American Institutrons for the Insane, commenced its third meeting, at the Astor House in the city of New York, on the 8th of May, 1848, the Vice President, William M. Awl, M. D., in the chair, and Thomas S. Kirkbride, M. D., Secretary.

Present—Dr. James Bates, of the Marine Insane Hospital, at Augusta; Dr. Andrew McFarland, of the New Hampshire State Hospital, at Concord; Dr. William H. Rockwell, of the Vermont State Hospital, at Brattleboro; Dr. Luther V. Bell, of the McLean Asylum for the Insane, at Somerville, Mass.; Dr. C. H. Stedman, of the Boston Lunatic Asylum; Dr. N. Cutter, of the Private Institution at Pepperill, Mass.; Dr. John S. Butler, of the Connecticut Retreat, at Hartford; Dr. Amariah Brigham, of the State Lunatic Asylum, Utica, N. York; Dr. Pliny Earle, of the Bloomingdale Asylum, N. Y.; Dr. James McDonald, of the Private Institution at Flushing, L. I.; Dr. —— Renney, of the Lunatic Asylum, on Blackwell's Island, N. Y.; Dr. G. H. White, of the Hudson (private) Lunatic Asylum, N. Y.; Dr. Horace A. Buttolph, of the New Jersey Lunatic Asylum, at Trenton; Dr. Thomas S. Kirkbride, of the Pennsylvania Hospital for the Insane, at Philadelphia; Dr. Joshua H. Worthington, of the Friends' Asylum, at Frankford, Pa.; Dr. N. C. Benedict, of the Blockley Insane Asylum, at Philadelphia; Dr. Fonerden, of the Maryland Hospital at

Baltimore; Dr. Wm. M. Awl, of the Ohio Lunatic Asylum, at Columbus; Dr. John M. Galt, of the Eastern Asylum, of Virginia, at Williamsburg; and Dr. John R. Allan, of the Kentucky Lunatic Asylum, at Lexington.

Dr. Samuel B. Woodward tendered his resignation of the Presidency of the Association, which was accepted, and Dr. Wm. M. Awl was elected President in the place of Dr. Woodward, resigned ; and Dr. A. Brigham, Vice President, in the place of Dr. Awl, elected President, The following preamble and resolutions, in reference to its late President, were unanimously adopted by the Associetion, viz:

WHEREAS, Dr. Samuel B. Woodward, at the present meeting of this Association, has tendered his resignation as President thereof.

*Resolved*, That whilst accepting this resignation, we cannot adjourn without declaring onr high sense of the services of Dr. Woodward as President of this body; and also, our full appreciation of his ardent and useful exertions, for so many years in behalf of the unfortunate insane.

*Resolved*, That the Secretary of the Association be requested to transmit to Dr. Woodward a copy of this resolution.

Agreeably to appointment, Dr. Brigham read an obituary notice of the late Dr. White, of the Hudson Lunatic Asylum, and the first Vice President of this Association, which was directed to be entered upon the minutes.

Dr. A. V. Williams and Dr. Benjamin Ogden, two of the visiting physians of the Asylum on Blackwell's Island, were invited to attend the sittings of the Association; and a resolution was adopted, authorizing each member to invite any person interested in its discussions.

In conformity with a resolution, adopted at the last meeting of the Association, Drs. Brigham and Macdonald made written, and Drs. Earle, Rockwell, Bates, Butler, Allan, and Kirkbride, verbal reports on the subjects of post-mortem examinations and the pathology of insanity, which, after consideration, were referred to the standing Committee on these subjects.

Dr. Kirkbride read a report from the committee on publication, which was accepted, and the Association subsequently

*Resolved*, That the committee on publication, appointed at the last meeting, be continued, and instructed to publish such of the reports, and such parts of the reports made to this Association, and snch parts of its proceedings as they shall deem conducive to the public good.

Elevations and ground plans of many of the institutions for the insane in the United States and Canada were laid upon the table for examination by the members of the Association;—also, a great variety of carving and fancy work, made by patients in the New York State Asylum,—and a number of ingenious bnckles and other improved fixtures, intended to be employed on restraining apparatus, and sent to the Association by the maker, John D. Fisher, Philadelphia.

Written reports were made, on the following subjects, and after full discussion, accepted and laid upon the table, subject to future disposition by the Association, viz:

On the comparative value of the different kinds of labor for patients, and the best means of employment in winter, by Dr. Rockwell; on the advantages and disadvantages of cottages for wealthy patients, adjacent to hospitals for the insane, by Dr. Kirkbride; on the relative value of the different kinds of fuel, for heating hospitals, by Dr. Bates; on the most economical mode of treating the insane of the poorer classes, by Dr. McFarland; on reading, recreations and amusements for the insane, by Dr. Galt; on the comparative valne of treatment in public institutions, and private practice, by Dr. White; and on the effects on the insane, of the use of tobacco, by Dr. Cutter.

Remarks on the diseases and causes of death among the insane, were also read by Dr. Macdonald; on the statistics of insanity, by Dr. Earle; and a series of cases of mania-a-potu, treated by the inhalation of ether, in the Boston City Hospital, by Dr. Stedman.

Invitations were received and accepted, to visit the Bloomingdale Asylum, under the care of Dr. Earle, and the private Institution at Flushing, Long Island, under the care of Dr. Macdonald; and both institutions were subsequently visited, and examined with great satisfaction, and the thanks of the Association tendered to these gentlemen for their courtesy, attention, and bountiful hospitality.

The Association also accepted an invitation to visit the Asylum on Blackwell's Island, and after a thorough examination of the buildings and arrangements, unanimously adopted the following resolutions:

*Resolutions respecting the Receptacle for Pauper-lunatics at Blackwell's Island.*

The "Association of Medical Superintendents of the American Institution for the Insane," holding their third biennial meeting in this city, have availed themselves of the kind invitation of the civil authorities superintending the receptacle for the pauper lunatics of this great metropolis, at Blackwell's Island, to visit and examine the unfortunate class there resident, and the provision made for their care, their amelioration, and their recovery.

It would be far more grateful to their feelings, could they leave this, as they do the other asylums for the insane, in this vicinity, which they have also examined, in silent but respectful regard at seeing great objects properly accomplished. In so doing, they would escape the unpleasant necessity of instituting painful criticisms in the face of personal civilities, and the hazard of being considered, by the unreflecting, as guilty of improper interference in the affairs of a community not their own.

Devoted, as most of them have been, for many long years of their lives, to the care and restoration of those deprived of reason; familiar, as many of them have been, from personal examination, with the condition of this class of sufferers, under the varying circumstances of the different communities of the old and new world; looking upon themseves, while citizens of widely separated states, yet common denizens of that republic of humanity that knows no state lines, they willingly venture all risk of being misunderstood and misrepresented, when they declare their conviction, that the arrangements for the three or four hundred pauper lunatics of this city, are far in the rear of the age, of the standard of other regions equally advanced in civilization and refinement, of the imperative demands of common justice, humanity and respect due to the image of a common Father, however much disfigured and changed.

They would, therefore, appeal to the authorities of this mighty and opulent metropolis of the Western world, to sustain the honor of their leading position; to those who must feel that they and their children have no immunity against loss of property, of friends, and of reason; to those who recognize the obligation imposed by their own elevation and success, to protect the friendless and miserable, to interpose their determined resolution, no longer to permit the Empire City to stand below the demands of the age, in the justice, humanity—yea, in the common decency, with which those guilty ol no crime, but stricken by the hand of Providence in the loss of reason are treated. Suffer no longer, we implore you, those whose sensibilities are not extinguished, but may even be more intense; whose honest self-respect and pride of character is not always permanently obliterated; whose return to society and to usefulness is not elsewhere the rare exception, but the expected result; to be abandoned to the tender mercies of thieves and prostitutes, who are, to a considerable extent, the associates and keepers of this helpless charge, and clothed with all the delegated authority and influence, which such a relation necessarily implies.

This Association has neither the means nor disposition to inquire why the pauper lunatics of this community should have been allowed to lapse into that depth of degradation and neglect, of which, it would be difficult, elsewhere, to find a parallel.

Enough is it for them to know, that such is the fact, notwithstanding plans and designs, for every modern architectural requirement, as well as curative and ameliorating appliances, have been long in the hands, and subject to the favorable criticism and comparison of those elsewhere charged with the same duties, and have been recognized as fully adequate to meet the exigency.

They have examined the recent report of the medical visitors, and conclude with them fully in their conclusions, as to the necessity of an entire change in the system; in the impossibility of doing all that justice, humanity, and a sound economy require for the insane, except at a cost of money sufficient to provide faithful, competent, respectable assistants or keepers, and adequate means of classification, inspection, labor, amusement, ventilation, and cleanliness. They believe a just economy requires the abandonment, or convertion to collateral uses merely, of those miserable apologies for insane hospitals, known as the old and the new *mad-houses;* and that, if the island is retained as a site for these institutions, the original design, fully satisfactory in its great outlines and principles, should at once, be carried out to completion.

The following preamble and resolution were adopted by the Association, viz:

WHEREAS, in the selection of medical superintendents to

American institutions for the insane, it is important to choose men with the highest qualifications, both as respects professional acquirements and moral endowments, therefore,

*Resolved*, That any attempt, in any part of this country, to select such officers through political bias, be deprecated by this Association, as a dangerous departure from that sound rule which should govern every appointing power, of seeking the best men, irresponsive of every other consideration.

The following resolutions were also adopted during the different sessions of the Association:

*Resolved*, That a committee be appointed to report to this Association at its next meeting, the best terms for the classification and designations of the different forms of insanity, and also the best anatomical and pathological terms for the various parts of the brain, and a nomenclature of the diseases which prove fatal to the insane.

*Resolved*, That a committe be appointed to suggest the best plan of calling the attention of physicians, in general practice, to the proper treatment of the insane at their homes, and especially to their treatment during the first period of their disease.

*Resolved*, That the members of this Association be requested to prepare and present to a future meeting, a statistical analysis of all the cases of insanity which have been admitted into the differvnt insitutions under their care.

*Resolved*, That all subjects heretofore referred to committees and not reported on at this meeting of the Association, be continued in the hands of the present committees for future action.

*Resolved*, That a committee be appointed, who shall, either before or after our adjournment, select subjects and appoint members to report on the same, in writing, at the next meeting of the Association.

*Resolved*, That previous to the future meetings of the Association, the Secretary be requested to invite the Boards of Trustees, managers, or official visitors of each insane asylum on this continent, to attend the sessions of this body.

*Resolved*, That the thanks of this Association be tendered to Messrs. Coleman & Stetson, of the Astor House, for their very liberal provision for the meetings of the Association, and for which, on account of its benevolent object, they have declined receiving compensation.

*Resolved*, That the thanks of the Association be tendered to the officers, for the able manner in which they have performed the duties of their respective stations.

*Resolved*, That the Secretary be instructed to furnish an abstract of the proceedings of the Association to the editor of the American Journal of Insanity, and to the editors of the various Medical Journals in the United States, for publication in their respective periodicals.

The Association continued its sessions until the afternoon of the 12th of May, and then adjourned to meet in the city of Utica, New York, on the 3d Monday of May, 1849, at 10 o'clock, A. M.

By order of the Association.

THOMAS S. KIRKBRIDE, *Secretary*.

